# Supplementary material for: Identification of Freezing-Responsive microRNAs and Their Targets in Chinese Jujube by Small RNA and Degradome Sequencing
Source: Int J Mol Sci. 2026 Sep 4;27(17):7912. doi: 10.3390/ijms27177912 (PMC13566890; doi:10.3390/ijms27177912)
Supplement: Supplementary file 1 [file ijms-27-07912-s001.zip › Supplementary Material S2/novel_miR_213.pdf]

Provisional ID	:	novel_miR_213
Score total	:	1515.6
Score for star read(s)	:	3.9
Score for read counts	:	1507.7
Score for mfe	:	2.4
Score for randfold	:	1.6
Score for cons. seed	:	
Total read count	:	2969
Mature read count	:	2829
Loop read count	:	116
Star read count	:	24

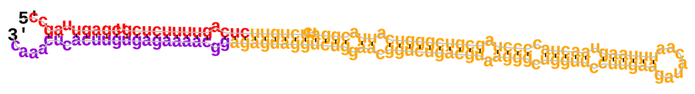

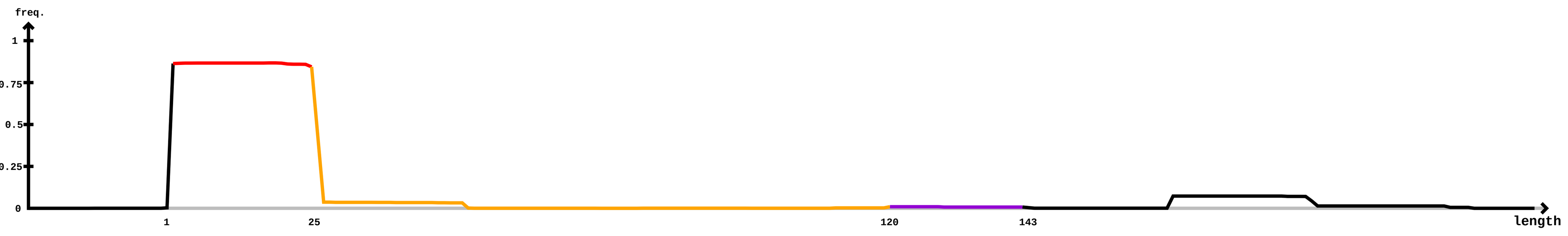

Mature Star

```
5'- uuacuggauacuaagaucaaguccgauugagcugcucuuuugacucuugucucuaggcauuacugggcugcgaucccccaucaaugaauuuaacauagaaguuccuuggucgggaaugcagucuggcaaggucuggaugagaggcaaaagaguguucacucaaacuuuuccuuggcuuccaauaacaacauugagcguucaacaucuuuagcaugccaccaugcaaaguccaauuuuuaaaggugguucca -3'  obs
    uuacuggauacuaagaucaaguccgauugagcugcucuuuugacucuugucucuaggcauuacugggcugcgaucccccaucaaugaauuuaacauagaaguuccuuggucgggaaugcagucuggcaaggucuggaugagaggcaaaagaguguucacucaaacuuuuccuuggcuuccaauaacaacauugagcguucaacaucuuuagcaugccaccaugcaaaguccaauuuuuaaaggugguucca      exp
    ....((((..(((((...((((..((.(((((.((((((((.((((((((..((((.((.(((((((((.((((.(((((.((((((......)))))).))))).)))).))))))))).)).))))))))))))).))))))))))))).))..))))...)))))..))))..........((((......((((((((((((....)))))).............))))))))))))..  reads  mm  sample
    .........acuaagaucaaguccgauugagcugcuc........................................................................................................................................................................................................................  1  0  LT-3
    .....................uccgauugagcugcucuuuugacuc...............................................................................................................................................................................................................  2  0  LT-1
    .....................uccgauugagcugcucuuuugacuc...............................................................................................................................................................................................................  1  0  LT-2
    .....................uccgauugagcugcucuuuugacuc...............................................................................................................................................................................................................  5  0  LCK-3
    ......................ccgauugagcugcucuuu.....................................................................................................................................................................................................................  5  0  LCK-3
    ......................ccgauugagcugcucuuuu....................................................................................................................................................................................................................  1  0  LT-2
    ......................ccgauugagcugcucuuuu....................................................................................................................................................................................................................  1  0  LT-1
    ......................ccgauugagcugcucuuuu....................................................................................................................................................................................................................  14  0  LCK-3
    ......................ccgauugagcugcucuuuug...................................................................................................................................................................................................................  5  0  LCK-3
    ......................ccgauugagcugcucuuuugac.................................................................................................................................................................................................................  2  0  LT-1
    ......................ccgauugagcugcucuuuugacA................................................................................................................................................................................................................  1  1  LT-1
    ......................ccgauugagcugcucuuuugacu................................................................................................................................................................................................................  1  0  JCK-3
    ......................ccgauugagcugcucuuuugacu................................................................................................................................................................................................................  1  0  JCK-2
    ......................ccgauugagcugcucuuuugacu................................................................................................................................................................................................................  1  0  LT-1
    ......................ccgauugagcugcucuuuugacu................................................................................................................................................................................................................  5  0  LT-3
    ......................ccgauugagcugcucuuuugacu................................................................................................................................................................................................................  3  0  LT-2
    ......................ccgauugagcugcucuuuugacu................................................................................................................................................................................................................  36  0  LCK-3
    ......................ccgauugagcugcucuuuugCcuc...............................................................................................................................................................................................................  2  1  LCK-1
    ......................ccgauugaAcugcucuuuugacuc...............................................................................................................................................................................................................  1  1  LCK-2
    ......................ccgauugCgcugcucuuuugacuc...............................................................................................................................................................................................................  1  1  LCK-3
    ......................ccgauNgagcugcucuuuugacuc...............................................................................................................................................................................................................  1  1  LCK-1
    ......................ccgauugagcugcucuuuugacuc...............................................................................................................................................................................................................  376  0  LCK-1
    ......................ccgauugagcugcucuuuugacuc...............................................................................................................................................................................................................  30  0  LT-2
    ......................ccgauugagcugcucuuuugacuc...............................................................................................................................................................................................................  1  0  JT-2
    ......................ccgauugagcugcucuuuugacuc...............................................................................................................................................................................................................  6  0  JCK-1
    ......................ccgauugagcugcNcuuuugacuc...............................................................................................................................................................................................................  2  1  LCK-1
    ......................ccgauugagcugcucCuuugacuc...............................................................................................................................................................................................................  1  1  LCK-3
    ......................ccgauugCgcugcucuuuugacuc...............................................................................................................................................................................................................  1  1  JCK-2
    ......................ccgauugagcugcucuuuugacuc...............................................................................................................................................................................................................  411  0  LCK-2
    ......................ccgauugagcugcucuuuugacuc...............................................................................................................................................................................................................  2  0  JT-3
    ......................ccgauugagcugcucuuuugacuc...............................................................................................................................................................................................................  4  0  JT-1
    ......................ccgauugagcugcucuuuugacuc...............................................................................................................................................................................................................  13  0  JCK-3
    ......................ccgauugagcugcucuuuugacuc...............................................................................................................................................................................................................  11  0  JCK-2
```

Mature Star

```
uuacuggauacuaagaucaaguccgauugagcugcucuuuugacucuugucucuaggcauuacugggcugcgauccccaucaaugaauuuaacauagaaguuccuuggucgggaaugcagucuggcaaggucuggaugagaggcaaaagaguguucacucaaacuuuuccuuggcuuccaauaacaacauugagcguucaacaucuuuagcaugccaccaugcaaaguccaauuuuuaaagguggguucca
......................ccgauugagcugcuNuuuugacuc............................................................................................................................................................................................ 2 1 LCK-1
......................ccgauugCgcugcucuuuugacuc............................................................................................................................................................................................ 1 1 LCK-2
......................ccgauugagcugcucuuCugacuc............................................................................................................................................................................................ 1 1 LCK-2
......................ccgauugagcugcucuuuugacuc............................................................................................................................................................................................ 65 0 LT-3
......................ccgauugagcugcucuuuugacuc............................................................................................................................................................................................ 1677 0 LCK-3
......................ccgauugagcugcucuuuugacuc............................................................................................................................................................................................ 111 0 LT-1
......................ccgauugagcugUucuuuugacuc............................................................................................................................................................................................ 7 1 LCK-3
......................ccgauugagcugcucuuuugCcuc............................................................................................................................................................................................ 1 1 LCK-3
......................ccgauugagcugcucuuuugCcuc............................................................................................................................................................................................ 1 1 LT-1
......................ccgauugagcugcucuuuugacucA........................................................................................................................................................................................... 5 1 LCK-3
......................ccgauugagcugcucuuuugacucA........................................................................................................................................................................................... 1 1 LT-3
......................ccgauugagcugcucuuuugacucA........................................................................................................................................................................................... 1 1 LT-2
.......................cgauugagcugcucuuuugacuc............................................................................................................................................................................................ 3 0 LCK-3
.......................cgauugagcugcucuuuugacuc............................................................................................................................................................................................ 1 0 LT-1
........................gauugagcugcucuuuugacucuug......................................................................................................................................................................................... 4 0 LCK-3
..........................uugagcugcucuuuugacu............................................................................................................................................................................................. 1 0 LCK-3
.....................................uuuugacucuugucucuag.................................................................................................................................................................................. 1 0 LT-3
......................................uuugacucuugucucuaggca............................................................................................................................................................................... 3 0 LT-3
..............................................uugucucuaggcauuacugg........................................................................................................................................................................ 4 0 LT-1
..............................................uugucucuaggcauuacugggc...................................................................................................................................................................... 2 0 LT-1
..............................................uugucucuaggcauuacugggcugc................................................................................................................................................................... 6 0 LT-1
..............................................uugucucuaggcauuacugggNugc................................................................................................................................................................... 1 1 LT-2
..............................................uugucucuaggcauuacugggcugc................................................................................................................................................................... 91 0 LCK-3
..............................................uuAucucuaggcauuacugggcugc................................................................................................................................................................... 1 1 LCK-3
..............................................uugucucuaggcauuacugggcugc................................................................................................................................................................... 3 0 LT-3
..............................................uugucucuaggcauuacugggcugcU.................................................................................................................................................................. 4 1 LCK-3
.......................................................................gauccccaucaaugaauuuaac............................................................................................................................................................. 1 0 LT-1
....................................................................................................uuccuuggucgggaaugc.................................................................................................................................... 2 0 LT-3
......................................................................................................................agucuggcaaggucuggaugagU............................................................................................................. 1 1 LT-1
....................................................................................................................................uggaugagaggcaaaaga.................................................................................................... 1 0 LT-1
....................................................................................................................................uggaugagaggcaaaaga.................................................................................................... 6 0 LCK-3
.............................................................................................................................................ggcaaaagaguguucacucaaac...................................................................................... 4 0 LT-2
.............................................................................................................................................ggcaaaagaguguucacucaaac...................................................................................... 15 0 LCK-3
.............................................................................................................................................ggcaaaagaguguucacucaaacA..................................................................................... 4 1 LCK-3
.............................................................................................................................................ggcaaaagaguguucacucaaacA..................................................................................... 1 1 LT-3
....................................................................................................................................................................uuuuccuuggcuuccaauaacaac.............................................................. 2 0 LT-1
............................................................................................................................................................................................auugagcguucaacaucuu........................................... 6 0 LT-2
............................................................................................................................................................................................auugagcguucaacaucuuuag........................................ 1 0 LT-3
............................................................................................................................................................................................auugagcguucaacaucuuuagc....................................... 12 0 LCK-2
............................................................................................................................................................................................auugCgcguucaacaucuuuagc....................................... 1 1 LCK-3
............................................................................................................................................................................................auugagcguucaacaucuuuagc....................................... 3 0 LT-1
............................................................................................................................................................................................auugagcguucaacaucuuuagc....................................... 18 0 LCK-1
............................................................................................................................................................................................auugagcguucaacaucuuuagc....................................... 10 0 LT-3
............................................................................................................................................................................................auugagcguucaacaucuuuagc....................................... 10 0 LT-2
............................................................................................................................................................................................auugagcguucaacaucuuuagc....................................... 8 0 JT-1
............................................................................................................................................................................................auugCgcguucaacaucuuuagc....................................... 1 1 LT-2
............................................................................................................................................................................................auugagcguucaacaucuuuagc....................................... 3 0 JCK-2
............................................................................................................................................................................................auugagcguucaacaucuuuagc....................................... 47 0 LCK-3
............................................................................................................................................................................................auugagcguucaacaucuuuagca...................................... 3 0 JCK-2
............................................................................................................................................................................................auugagcguucaacaucuuuagca...................................... 2 0 JCK-3
............................................................................................................................................................................................auugagcguucaacaucuuuagca...................................... 99 0 LCK-3
............................................................................................................................................................................................auugagcguucaacaucuuuagca...................................... 2 0 JCK-1
............................................................................................................................................................................................auugagcguucaacaucuuuagca...................................... 5 0 LT-1
............................................................................................................................................................................................auugagcguucaacaucuuuagca...................................... 7 0 LT-3
...................................................................................................................................................................................................................augccaccaugcaaaguccaauu................ 4 0 JT-3
...................................................................................................................................................................................................................augccaccaugcaaaguccaauu................ 1 0 LT-3
...................................................................................................................................................................................................................augccaccaugcaaaguccaauu................ 3 0 LT-1
...................................................................................................................................................................................................................augccaccaugcaaaguccaauu................ 16 0 LCK-3
...................................................................................................................................................................................................................augccaccaugcaaaguccaauu................ 2 0 JT-2
....................................................................................................................................................................................................................ugccaccaugcaaaguccaauu................ 1 0 LT-1
....................................................................................................................................................................................................................ugccaccaugcaaaguccaauuuuua............ 4 0 LCK-3
....................................................................................................................................................................................................................ugccaccaugcaaaguccaauuuuua............ 7 0 LCK-1
....................................................................................................................................................................................................................ugccaccaugcaaaguccaauuuuua............ 6 0 LCK-2
....................................................................................................................................................................................................................ugccaccaugcaaaguccaauuuuua............ 1 0 LT-3
```
